# Comparative Analysis of Old-Age Mortality Estimations in Africa

**DOI:** 10.1371/journal.pone.0026607

**Published:** 2011-10-19

**Authors:** Eran Bendavid, Benjamin Seligman, Jessica Kubo

**Affiliations:** 1 Division of General Internal Medicine, Stanford University, Stanford, California, United States of America; 2 Center for Health Policy and the Center for Primary Care and Outcomes Research, Stanford University, Stanford, California, United States of America; 3 School of Medicine, Stanford University, Stanford, California, United States of America; University of Otago, New Zealand

## Abstract

**Background:**

Survival to old ages is increasing in many African countries. While demographic tools for estimating mortality up to age 60 have improved greatly, mortality patterns above age 60 rely on models based on little or no demographic data. These estimates are important for social planning and demographic projections. We provide direct estimations of older-age mortality using survey data.

**Methods:**

Since 2005, nationally representative household surveys in ten sub-Saharan countries record counts of living and recently deceased household members: Burkina Faso, Côte d'Ivoire, Ethiopia, Namibia, Nigeria, Swaziland, Tanzania, Uganda, Zambia, and Zimbabwe. After accounting for age heaping using multiple imputation, we use this information to estimate probability of death in 5-year intervals (_5_q_x_). We then compare our _5_q_x_ estimates to those provided by the World Health Organization (WHO) and the United Nations Population Division (UNPD) to estimate the differences in mortality estimates, especially among individuals older than 60 years old.

**Findings:**

We obtained information on 505,827 individuals (18.4% over age 60, 1.64% deceased). WHO and UNPD mortality models match our estimates closely up to age 60 (mean difference in probability of death -1.1%). However, mortality probabilities above age 60 are lower using our estimations than either WHO or UNPD. The mean difference between our sample and the WHO is 5.9% (95% CI 3.8–7.9%) and between our sample is UNPD is 13.5% (95% CI 11.6–15.5%). Regardless of the comparator, the difference in mortality estimations rises monotonically above age 60.

**Interpretation:**

Mortality estimations above age 60 in ten African countries exhibit large variations depending on the method of estimation. The observed patterns suggest the possibility that survival in some African countries among adults older than age 60 is better than previously thought. Improving the quality and coverage of vital information in developing countries will become increasingly important with future reductions in mortality.

## Introduction

Mortality estimates and life tables for many developing countries are associated with great uncertainty [1]. In developed countries, mortality is monitored with vital registration systems and population censuses. In developing countries, however, vital registration systems are mostly non-existent and censuses are rare [2]. As a result, mortality estimations and life tables published by the United Nations Population Division (UNPD) and World Health Organization (WHO) for most developing countries rely on demographic models [3], [4]. These models use limited available vital information such as survey-based data on child mortality to estimate survival patterns for the entire population [5], [6]. This is done by tying the available mortality data to historical patterns obtained in developed countries, making the implicit assumption that mortality patterns in developing countries today fall within the range of historical mortality patterns observed in developed countries.

The uncertainty in mortality estimations is particularly large for older ages. Up to age 60, the increasing availability of nationally representative, high-quality survey data has enabled direct measurements of child and adult mortality; however, in many countries no nationally-representative mortality data is available among individuals older than 60 years-old [7], [8]. This information on the health of the elderly is important for social policies, demographic projections, and research [9], [10]. The absence of data and the use of models that rely on historical data from developed countries makes old-age mortality particularly uncertain. This issue is further complicated by the HIV epidemic's distortion of mortality patterns in many African countries. Our current knowledge suggests that the heaviest mortality burden of the HIV epidemic is shared by men and women between 15 and 49 years old [11]. The burden of HIV among prime-aged adults could affect old-age survival through the declining support for Africa's elderly [12]. The epidemic could also create artefactual distortions in old-age mortality models. For example, old-age mortality could appear high if mortality among adults older than 60 is extrapolated from survival at younger ages. Some demographic models incorporate HIV burden in adjusting demographic estimates, but the burden of the epidemic among the elderly is poorly characterized: HIV sero-surveillance is rarely done on individuals older than 50, and prevalence among Africa's elderly is poorly understood[13]. All these issues contribute to the difficulties associated with mortality estimates among Africa's elderly.

We explore a new approach for estimating survival at older ages in ten African countries using new Demographic and Health Surveys (DHS) mortality data collected since 2005. We compare mortality patterns between those implied by the newly available DHS data and those reported by UNPD and WHO. We show a pattern of differences between modeled and measured mortality, estimate the demographic and epidemiologic implications of these findings, explore the reasons for the differences, and propose future improvements to approaches for estimating old-age mortality.

## Methods

### DHS Household Mortality Data

We use person-level household census and mortality data in the DHS. These surveys, conducted every 5 to 6 years in many developing nations, provide nationally representative samples of between 3,000 and over 30,000 households [14]. This data is commonly considered among the highest quality demographic data available for many developing countries, and it is the primary source for estimating child mortality, adult mortality, and maternal mortality in many developing countries [7], [8], [15]. DHS household schedules enumerate all the usual residents of the household along with their age and gender. Since 2005, DHS surveys in eight African countries included a household mortality module. The module, “Support for People Who Have Died,” was implemented in countries with high HIV burden to collect information on services such as medical, psychological, material, or social assistance to ailing and recently deceased household members [16]. Information on gender and age at death is recorded for all deaths in a household in the past 12 months. While this module was not collected for demographic purposes, it provides a representative sample of vital information on old-age mortality. To the best of our knowledge, this is the first nationally representative data tracking mortality in this population using a consistent methodology across multiple countries. We combine this information on mortality together with household membership to estimate age-specific mortality patterns.

While the household census module is available in every DHS, mortality information is only available for Cote d'Ivoire (2005), Namibia (2006–7), Nigeria (2008), Swaziland (2006–7), Tanzania (2007–8), Uganda (2006), Zambia (2007), and Zimbabwe (2005–6). In addition, the Global Fund to Fight AIDS, TB and Malaria (Global Fund) conducted nationally representative household surveys with identical mortality modules (taken verbatim from DHS) in three countries as part of its 5-year evaluation in 2007 and 2008: Ethiopia, Burkina Faso, and Zambia. In the end, we analyzed data from ten countries, with one country's data (Zambia) available from two similar surveys. We refer to all survey-based mortality data as DHS, regardless of whether it was collected by the Global Fund or DHS.

### Survey Data Manipulation and Smoothing Age Heaping

The household survey is structured to contain one observation per household. Within that observation is a record of all the people alive in the household, their gender, and age, as well as the age at death and gender of all who died in the household within the past 12 months. From this data we extracted information on the population alive and deceased in 1-year age intervals for the year preceding the survey. Age-heaping, or the tendency to report ages in multiples of 5 or 10 years, is a known concern with age estimations in DHS [17], [18]. and we applied a smoothing algorithm to distribute heaped ages [19]. Heaped ages were redistributed using a uniform distribution centered at the age with a five year window. Thus, an individual who reported his or her age as X was reassigned to neighboring ages with a uniform distribution U(X−5, X+5). Ages were imputed five times, specific to each country and gender, as age heaping was marked in some countries and less noticeable in others.

### Existing WHO and UNPD Mortality Estimates

Several agencies produce international compilations of demographic data, including the World Bank and the US Census Bureau. Among those, the WHO and UNPD update their products regularly. WHO produces life tables as the outcome of interest, while UNPD compiles and models fertility, mortality, and population data. Their data and methods differ, especially for old-age mortality estimations where vital registration is absent. The WHO uses a modification of the Brass logit approach that assumes age-structured mortality is a linear transformation of the logit of a standard life table. In most low- and middle-income countries (LMIC), DHS is used as a primary data source to estimate the two parameters needed for the Brass transformation: l_5_ and l_60_ (probability of survival to age 5 and 60, respectively). The accuracy of this linear transformation assumption was recently tested and found to be reasonable only up to age 60 [20]. The UNPD uses variations of the Coale-Demeny and United Nations methods using DHS and census data where available [21], [22], [23], [24]._ENREF_21 UNPD's primary output is population size, and it provides ranges and uncertainty associated with future population projections, but only a single mortality estimate.

### Demographic Estimates and Analyses

The household mortality and census counts allowed us to estimate the age-specific mortality rates. We grouped the counts into 5-year intervals starting at age 15 and going up to age 95 and above (denoted 95+). We use standard assumptions to calculate the probability of death in each interval, denoted _n_q_x_ for the interval starting at age x and ending at x+n [25]. Specifically, we calculated the age-specific mortality rate, _n_m_x_, as the ratio of age-specific deaths to population size, and estimated _n_q_x_  = (n*_ n_m_x_)/(1+_ n_m_x_*(n-_ n_a_x_)), where _n_a_x_ is the average length of time lived among those who died in the interval from x to x+n.

Handling bias is a challenge when estimating mortality from survey data. Mortality under-reporting has been documented elsewhere, and may be introduced if older individuals die outside the home, if households with high mortality rates are selected against in the sampling scheme because they only have a few living family members left alive (if any), and if cultural factors affect death reporting, especially at old ages [26], [27]. One correction for this bias involves weighting for within-household mortality (the ratio of those who died to those who survived at the time of the survey) and accounting for households in which all members had died [27]. We find that over 98% of households report zero or one death and the ratio of those who died to those currently residing in the house ranges from 0 to 0.1 in over 95% of households, suggesting this weighting parameter will not alter the underlying data substantively. Unlike the sibling survival module which records the history of the index woman's entire sibship and which Gakidou and King address, the module used in this analysis records deaths only within the household and only in the past 12 months. That is, only a small proportion of household members have died in the span of a year, and only a few households have decreased to near-zero survivors.

We then compare our age-specific mortality estimates to widely published estimates from the WHO and UNPD. WHO provides complete life tables (including _n_q_x_) for all member states, while the UNPD provides information on survivorship to age x, denoted *l_x_* . We convert the probability of dying in the interval of length n years (from x to x+n) by _n_q_x_  =  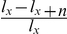.

We calculate differences in age-specific probabilities of death between the modeled WHO and UNPD estimates and those obtained from DHS data. We then estimate the difference in the expectation of remaining life between the UNPD/WHO estimates and our measured estimates (assuming mortality patterns have not changed substantially for this age group in the past five years) for the current cohort of 60-year old men and women in each country. From this we can estimate the gap in older-age life years between official estimates and our estimates.

## Results

Our primary data consisted of eight DHS surveys and 2 Global Fund surveys from 2005 to 2008. The surveys provided information on a total of 93,734 households, 497,682 living household members, and 8,145 recently deceased members. Women represented 47% of deaths and 51% of those alive. Table 1 provides more details on the study population.

**Table 1:** Survey countries, years, and characteristics.

Country (year)	Households	Individuals	
		Alive	Recent deaths
Burkina Faso (2007)	2,425	44,869	1,095
Côte d'Ivoire (2005)	4,368	24,920	287
Ethiopia (2008)	5,012	37,816	652
Namibia (2006–7)	9,200	42,560	670
Nigeria (2008)	34,070	156,541	2,471
Swaziland (2006–7)	4,843	22,128	509
Tanzania (2007–8)	8,497	45,182	432
Uganda (2006)	8,870	45,428	661
Zambia (2007)	7,164	35,555	505
Zimbabwe (2005–6)	9,285	42,683	863

**Figure 1:**
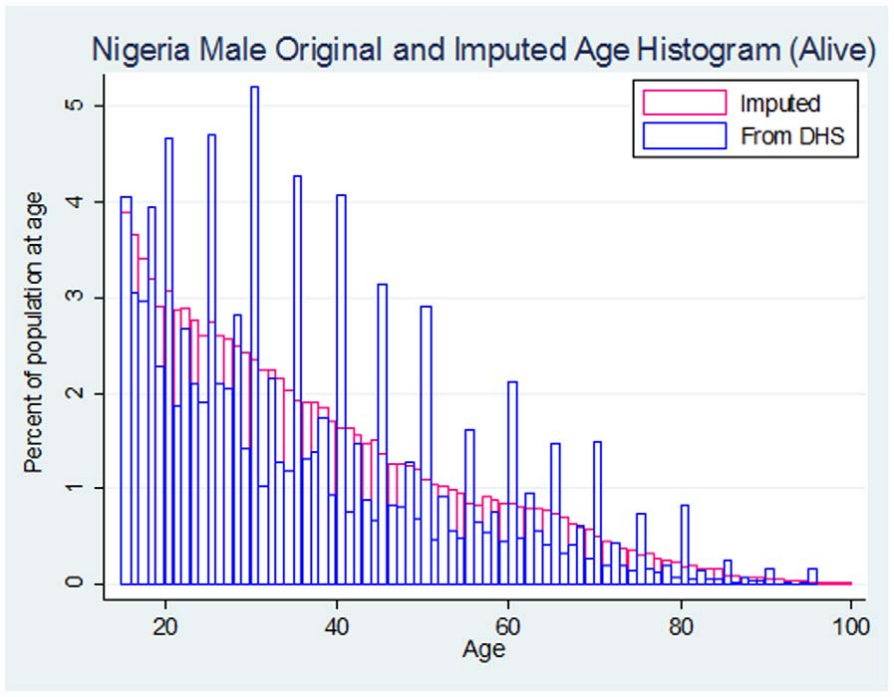
Survey age distribution and imputed age distribution for living male household members in Nigeria. Evidence of age heaping among male Nigerian household members, and the effect of smoothing. Heaping is apparent in ages that are multiples of 5 (worse on even multiples than odd). Smoothing was accomplished by redistributing the frequency at each age to neighboring age categories with a uniform distribution.

Heaping was apparent for age reporting of both living and recently deceased household members. This was more pronounced at ages that are multiples of ten than odd multiples of five. In Nigeria, for example, the ratio between the number of living men reported to be 30, 40, 50, or 60 years old and the number of people 1-year away (e.g. 29, 31, 39, 41, etc.) was 4.6. We smoothed the reported age distribution based on the multiple imputation technique described above. Figure 1 overlays the reported age distribution of living men in Nigeria on top of the same population's smoothed age distribution. Imputed age distributions were used for all remaining analyses.

**Figure 2:**
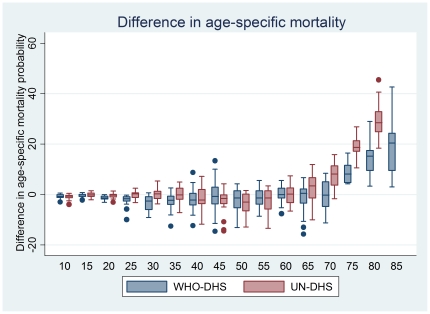
Differences in age-specific mortality. Differences in age-specific mortality (_n_q_x_) between the UNPD/WHO estimates and survey-based (DHS) estimates. For each age group, we obtained a difference between the modeled (WHO in blue, UNPD in red) and DHS-based probability of death for each of the study's ten countries, separated by gender. Variance increases with age due to the smaller population sizes. Above age 60, the mean difference starts rising, and continues to rise all the way to the highest age bracket.

Comparisons of mortality probabilities between WHO, UNPD and our estimates show that the probability of death provided by WHO and UNPD are nearly always higher than our estimates above age 60. This discrepancy is not seen in ages 60 and younger. This is shown in Table 2, Table S1, and Figure 2. Among men and women in the age interval from 55 to 59, the median difference in probability of death between WHO and DHS estimates is -1.4% (IQR -3.9 - 1.5%) and -1.3% (IQR -5.8 - 1.5%) using UNPD data. However, among men and women in the age interval from 75 to 79, this probability of death is greater than the DHS estimates by 5.4% (IQR 2.1–10.0%) in the WHO estimates and 17.6% (IQR 13.6–20.2%) in the UNPD estimates. The difference between the modeled and observed mortality estimates begins to grow only after age 60 and continues to grow with older ages. We regress age as a categorical predictor of the difference between the WHO/UNPD estimates and those from DHS, and observe significant overestimations of mortality starting at age 65 (for UNPD) and 70 (for WHO). The age-specific probability of death between ages 75 and 80 years old is higher than the observed mortality by 0.24 in the UNPD estimates (0.20–0.29, p<0.001) and by 0.11 (0.04–0.18, p = 0.002) in the WHO estimates. These estimates by age group are shown in Table 3. On average, the difference in probability of death in any 5-year period above age 60 is 5.9% (95% CI 3.8–7.9%) in comparison with WHO data and 13.5% (95% CI 11.6–15.5%) compared with UNPD using 2-tailed t-tests.

**Table 2:** Age-specific probability of death by method of estimation in the ten study countries.

	60–64			65–69			70–74		
	Study	WHO	UNPD	Study	WHO	UNPD	Study	WHO	UNPD
Burkina Faso M	13.7	12.8	16.7	14.5	16.8	26.4	29.8	22.8	39.7
Burkina Faso F	11.3	9.9	13.5	13.1	12.8	21.9	30.4	19.2	34.0
Côte d'Ivoire M	9.2	11.3	11.0	11.7	14.7	18.2	16.4	20.8	29.0
Côte d'Ivoire F	8.8	8.5	7.1	10.6	11.2	12.7	8.4	16.8	22.0
Ethiopia M	8.1	10.8	12.0	13.3	14.5	18.6	19.4	20.5	27.9
Ethiopia F	2.3	7.9	9.7	8.6	10.7	15.4	17.5	16.2	23.7
Namibia M	17.3	9.7	10.7	25.6	12.6	17.1	27.9	17.7	26.3
Namibia F	7.0	5.4	7.1	8.5	7.3	12.1	11.3	10.9	20.1
Nigeria M	8.6	13.3	13.9	10.3	17.0	21.2	15.4	23.2	31.2
Nigeria F	7.3	10.6	11.8	9.1	13.3	18.2	13.6	19.5	27.2
Swaziland M	17.8	12.6	13.6	30.7	15.0	20.7	27.8	19.6	30.6
Swaziland F	14.0	10.0	9.2	10.6	11.0	15.0	16.0	13.7	23.7
Tanzania M	9.4	14.2	11.2	18.1	17.6	17.4	20.8	23.3	26.3
Tanzania F	8.4	10.8	8.7	13.2	13.9	14.0	12.0	18.8	22.0
Uganda M	16.2	13.4	12.4	22.0	16.3	19.0	21.9	21.9	28.3
Uganda F	9.1	9.5	9.6	12.8	12.0	15.3	20.1	17.5	23.6
Zambia M	16.5	15.5	13.9	16.3	18.4	21.1	20.8	23.2	31.1
Zambia F	11.2	10.0	10.6	18.7	12.2	16.5	11.6	17.3	25.2
Zimbabwe M	18.2	20.7	13.4	27.8	17.2	20.3	28.5	21.6	29.9
Zimbabwe F	10.6	10.9	8.0	9.9	12.2	13.1	17.2	16.3	20.8

	75–79			80–84		
	Study	WHO	UNPD	Study	WHO	UNPD
Burkina Faso M	38.2	32.0	55.7	40.8	44.2	71.6
Burkina Faso F	52.8	28.2	49.4	85.2	39.9	66.0
Côte d'Ivoire M	32.2	29.8	43.8	52.3	41.8	60.8
Côte d'Ivoire F	20.2	25.4	35.8	8.2	37.2	53.7
Ethiopia M	13.2	29.7	40.0	28.6	41.8	54.3
Ethiopia F	18.8	24.9	35.1	30.7	36.7	49.2
Namibia M	17.3	25.7	38.7	30.9	37.1	53.6
Namibia F	12.7	17.4	31.9	14.6	28.1	47.5
Nigeria M	21.0	32.6	43.9	31.3	44.8	58.2
Nigeria F	15.9	28.6	39.1	24.1	40.2	53.0
Swaziland M	22.2	26.5	43.3	45.7	37.7	57.7
Swaziland F	24.2	20.2	35.9	15.6	30.8	51.1
Tanzania M	18.9	32.1	38.1	26.0	43.5	52.4
Tanzania F	22.6	26.9	33.2	22.4	37.8	47.3
Uganda M	21.8	31.2	40.5	21.6	43.2	54.7
Uganda F	18.0	26.2	35.0	20.8	37.9	49.2
Zambia M	26.3	31.9	43.7	17.4	43.4	58.0
Zambia F	26.1	26.0	36.8	28.4	37.9	51.0
Zimbabwe M	24.7	29.0	42.3	35.8	40.0	56.9
Zimbabwe F	13.2	23.8	32.0	13.5	34.9	46.4

**Table 3:** Association between modeled and observed mortality estimations by age*.

Age	WHO-DHS	p	UNPD-DHS	p
20–24	-0.009	0.71	0.002	0.89
25–29	-0.019	0.45	0.005	0.79
30–34	-0.017	0.48	0.002	0.92
35–39	-0.007	0.77	-0.012	0.52
40–44	0.004	0.87	-0.023	0.19
45–49	-0.006	0.82	-0.031	0.08
50–54	0.001	0.96	-0.021	0.23
55–59	0.015	0.56	0.003	0.85
60–64	-0.005	0.84	0.024	0.17
65–69	0.007	0.77	0.079	<0.001
70–74	0.045	0.07	0.158	<0.001
75–79	0.101	<0.001	0.248	<0.001
80–84	0.126	<0.001		

*All comparison tests done using 2-tailed t-tests.

One direct implications of these findings is that the remaining life expectancy of today's older population in the study countries is greater than that projected by WHO and UNPD. We estimate the gap in the expected remaining life years between WHO, UNPD, and DHS for the 65-year-old population in each country. We find that the WHO underestimates remaining life expectancy on average by 1.1 years and UNPD by 2.8 years on average for men, and 1.3 and 2.3 years (respectively) for women. This represents a substantial proportion of the remaining life expectancy, 10.4% compared with WHO and 14.4% compared with UNPD. By contrast, the difference in life expectancy as a proportion of the remaining life expectancy between ages 20 and 60 represents 3.4% when compared with the WHO and 4.8% when compared with UNPD.

## Discussion

We present estimates of older-age mortality in Africa that are based on nationally representative health surveys and capture survivorship and mortality at the household level. Our results suggest a pattern of differences between our estimates and those provided UNPD and WHO for adults older than 60 years-old, though not for younger adults. To the best of our knowledge, this is the first use DHS's household census data for mortality estimation. While the underlying data is limited by sampling errors and selection bias, the pattern is consistent across countries and limited to those ages where the WHO and UNPD are particularly uncertain. If true, these findings have three important implications: social planning that addresses resources for older individuals may need to be revised; generalizing historical mortality experience from developed countries may not appropriate for Africans older than 60 years today; and while the HIV epidemic has dramatically increased mortality in the younger African population, it may have spared the older population, at least until now.

A leading contribution of this work is to highlight the differences in old-age mortality estimations in countries where a vital registration system is absent. DHS survey methodology is reliable, its sampling schema transparent, and its ability to collect information from the most remote areas of the world's least developed countries unmatched. At the same time, DHS surveys only a small fraction of each country's households, it is not set up for ongoing collection of vital information, and the specific module we use has only been implemented recently. On balance, it is unclear whether DHS underestimates old-age mortality or the UN-based models overestimate it.

Regardless of the direction of the bias, the differences in old-age mortality estimates are substantial. These differences are noticeable between the UNPD and WHO estimates (that is, even without the DHS-based estimations). Our DHS-based estimates augment the magnitude of those differences. Uncertainty in mortality estimates for older individuals stems from the data sources underlying the WHO and UNPD models, the model assumptions, and DHS's sampling error in this age group. WHO anchors their Brass logit linear transformation at ages 5 and 60 using the best available data for these ages. That is, mortality estimates beyond age 60 are based solely on the shape of the mortality function. UNPD goes through a similar process, but rely on the Coale-Demeny and United Nations set of models instead of the Brass logit. The absence of underlying data for old-age mortality implies an inherent uncertainty in the UNPD and WHO models. On the DHS side, the number of elderly alive and deceased in the past 12 months can be relatively small even with surveys of several thousands of households and an average household size greater than four people. Taken together, our work highlights the inadequacy of existing methods for old-age mortality estimations across Africa.

Although the relative size of the over-60 population in most sub-Saharan countries is estimated at around 5% of the total population, that population is commonly dependent, growing in size, and its longevity, as our study suggests, is uncertain. Few African countries have social safety net programs for older individuals, and caring for the elderly is usually assumed to be the responsibility of family members. Our findings suggest the possibility that burden of caretaking for older kin on the younger population may be higher than expected. This has significant policy implications for planners who examine resource utilization for the elderly balanced against the economic growth enabled by reducing the dependence of the elderly on the younger population. The elderly may also be a source of assets and care for adult children who have HIV or orphaned grandchildren; thus, improved elder survival may also have positive welfare consequences.

Our findings also highlight the limitations of current methodologies used to estimate mortality patterns in countries lacking vital registration systems. Without count data of those alive and deceased, the best available approaches rely on historical experience from developed countries with census and mortality count data from decades or centuries ago. Because fertility and child survival patterns correlate well between today's Africa and historical Europe, it is then assumed that older-age survival correlates as well. Our analysis suggests that survival among Africa's elderly today is different than Europe's historical elderly.

Vital registration systems would obviate many of the issues brought up by our analysis. Establishing institutions that provide high-quality, high-coverage mortality and survivorship count data would address much of the uncertainty we highlight. Complete vital registration systems are costly and require documentation such as birth and death certificates, an unlikely goal for most African countries in the near term. However, hybrid approaches such as India's sample registration system which provides ongoing enumeration in a sample of the country's villages and towns are potentially feasible even in the absence of other infrastructure. Our study also suggests that the challenges and costs entailed in improving national vital statistics could be offset by the need for improved information on old-age mortality for social planning.

### Limitations

Several observations may bias our results towards lower-than-expected old-age mortality. First, while DHS are nationally representative and large by African standards, the death counts are low, especially at older ages. Over eight countries we had 1,233 deaths above age 60 (just under 20% of all deaths), or about 75 deaths per country and gender. Our uncertainty intervals are correspondingly wide. In addition, it is possible that the household surveys under-estimate old-age mortality because households where only elderly live or where all had deceased were not sampled, because of selective under-reporting of elder mortality, or because of surveyor preference for recording deaths below age 60. Our current surveys cannot account for these unobserved effects, but replicating the surveys and adding households where no younger adults live to the sampling scheme can reduce these biases. Finally, the gaps relative to the UNPD may be overestimated because the models used in its World Population Prospects 2008, which we use, have been revised to improve the impact of the HIV epidemic on mortality. The public release of the World Population Prospects 2010 did not include the dada needed for this analysis.

### Conclusion

Accurate demographic information is a cornerstone of research and policy planning, and its absence is many sub-Saharan countries is considered a first-order problem. We estimate mortality using a new module that collects information on household membership and recent deaths. Though we find consistently lower mortality than WHO and UNPD at older ages, the data underlying our findings is limited. Regardless, however, the large differences in old-age mortality suggest the need for future expansion of vital data collection.

## Supporting Information

Table S1 (DOCX)
