# Supplementary material for: Comparative Analysis of Old-Age Mortality Estimations in Africa
Source: PLoS One. 2011 Oct 19;6(10):e26607. doi: 10.1371/journal.pone.0026607 (PMC3197519; doi:10.1371/journal.pone.0026607)
Supplement: Table S1 — (DOCX) [file pone.0026607.s001.docx]

Appendix Table 1: Age-specific probability of death (_n_q_x_) by method of estimation for ages younger than 60

| Country | Age Group | Male (=1) | _n_q_x_ DHS | _n_q_x_ WHO | _n_q_x_ UNPD |
| --- | --- | --- | --- | --- | --- |
| Burkina Faso | 15 | 0 | 1.7 | 1.2 | 1.3 |
| Burkina Faso | 15 | 1 | 2.8 | 0.9 | 1.5 |
| Burkina Faso | 20 | 0 | 4.9 | 1.8 | 1.8 |
| Burkina Faso | 20 | 1 | 3.3 | 0.8 | 2.2 |
| Burkina Faso | 25 | 0 | 5.2 | 2.6 | 2.0 |
| Burkina Faso | 25 | 1 | 3.4 | 1.8 | 2.4 |
| Burkina Faso | 30 | 0 | 5.6 | 3.2 | 2.1 |
| Burkina Faso | 30 | 1 | 5.1 | 2.4 | 2.6 |
| Burkina Faso | 35 | 0 | 6.7 | 3.7 | 2.5 |
| Burkina Faso | 35 | 1 | 5.4 | 3.4 | 3.1 |
| Burkina Faso | 40 | 0 | 5.7 | 4.2 | 3.2 |
| Burkina Faso | 40 | 1 | 7.0 | 4.7 | 4.0 |
| Burkina Faso | 45 | 0 | 7.3 | 5.0 | 4.4 |
| Burkina Faso | 45 | 1 | 7.6 | 5.6 | 5.4 |
| Burkina Faso | 50 | 0 | 8.1 | 5.9 | 6.1 |
| Burkina Faso | 50 | 1 | 13.8 | 6.7 | 7.6 |
| Burkina Faso | 55 | 0 | 8.8 | 7.2 | 8.8 |
| Burkina Faso | 55 | 1 | 13.4 | 8.9 | 10.9 |
| Cote dIvoire | 15 | 0 | 1.5 | 0.9 | 0.7 |
| Cote dIvoire | 15 | 1 | 2.9 | 0.9 | 0.8 |
| Cote dIvoire | 20 | 0 | 1.9 | 1.1 | 1.1 |
| Cote dIvoire | 20 | 1 | 2.9 | 0.9 | 1.1 |
| Cote dIvoire | 25 | 0 | 4.0 | 1.8 | 2.5 |
| Cote dIvoire | 25 | 1 | 2.9 | 1.6 | 1.8 |
| Cote dIvoire | 30 | 0 | 4.1 | 3.0 | 4.6 |
| Cote dIvoire | 30 | 1 | 2.2 | 2.1 | 3.1 |
| Cote dIvoire | 35 | 0 | 10.5 | 4.3 | 5.8 |
| Cote dIvoire | 35 | 1 | 5.2 | 3.0 | 4.7 |
| Cote dIvoire | 40 | 0 | 9.2 | 5.3 | 5.3 |
| Cote dIvoire | 40 | 1 | 7.6 | 4.8 | 5.7 |
| Cote dIvoire | 45 | 0 | 4.2 | 5.9 | 4.1 |
| Cote dIvoire | 45 | 1 | 6.9 | 6.0 | 6.0 |
| Cote dIvoire | 50 | 0 | 8.0 | 6.1 | 3.7 |
| Cote dIvoire | 50 | 1 | 10.5 | 6.2 | 6.4 |
| Cote dIvoire | 55 | 0 | 6.0 | 6.5 | 4.4 |
| Cote dIvoire | 55 | 1 | 16.9 | 8.3 | 7.5 |
| Ethiopia | 15 | 0 | 1.9 | 0.7 | 1.5 |
| Ethiopia | 15 | 1 | 2.9 | 0.7 | 1.7 |
| Ethiopia | 20 | 0 | 2.1 | 1.0 | 1.9 |
| Ethiopia | 20 | 1 | 2.7 | 0.9 | 2.4 |
| Ethiopia | 25 | 0 | 2.1 | 1.5 | 2.6 |
| Ethiopia | 25 | 1 | 4.0 | 1.5 | 2.8 |
| Ethiopia | 30 | 0 | 3.4 | 2.1 | 3.7 |
| Ethiopia | 30 | 1 | 4.8 | 1.8 | 3.4 |
| Ethiopia | 35 | 0 | 4.9 | 2.8 | 4.4 |
| Ethiopia | 35 | 1 | 4.7 | 2.5 | 4.2 |
| Ethiopia | 40 | 0 | 7.5 | 3.4 | 4.6 |
| Ethiopia | 40 | 1 | 8.9 | 3.6 | 5.0 |
| Ethiopia | 45 | 0 | 5.3 | 3.9 | 4.5 |
| Ethiopia | 45 | 1 | 8.1 | 4.5 | 5.8 |
| Ethiopia | 50 | 0 | 5.3 | 4.6 | 5.0 |
| Ethiopia | 50 | 1 | 9.9 | 5.5 | 6.8 |
| Ethiopia | 55 | 0 | 4.3 | 5.7 | 6.6 |
| Ethiopia | 55 | 1 | 10.1 | 7.6 | 8.5 |
| Namibia | 15 | 0 | 1.1 | 0.3 | 0.3 |
| Namibia | 15 | 1 | 1.2 | 0.5 | 0.6 |
| Namibia | 20 | 0 | 2.1 | 0.4 | 1.1 |
| Namibia | 20 | 1 | 1.0 | 0.8 | 0.9 |
| Namibia | 25 | 0 | 6.9 | 1.1 | 3.8 |
| Namibia | 25 | 1 | 3.8 | 1.4 | 2.1 |
| Namibia | 30 | 0 | 10.0 | 2.5 | 6.9 |
| Namibia | 30 | 1 | 7.8 | 1.7 | 4.2 |
| Namibia | 35 | 0 | 8.5 | 4.3 | 7.6 |
| Namibia | 35 | 1 | 10.1 | 3.1 | 6.1 |
| Namibia | 40 | 0 | 10.1 | 5.2 | 6.0 |
| Namibia | 40 | 1 | 13.7 | 5.5 | 6.8 |
| Namibia | 45 | 0 | 15.0 | 5.4 | 4.2 |
| Namibia | 45 | 1 | 20.5 | 5.9 | 6.5 |
| Namibia | 50 | 0 | 10.2 | 4.7 | 3.6 |
| Namibia | 50 | 1 | 19.3 | 6.3 | 6.4 |
| Namibia | 55 | 0 | 5.5 | 4.4 | 4.5 |
| Namibia | 55 | 1 | 16.6 | 8.1 | 7.5 |
| Nigeria | 15 | 0 | 2.0 | 1.3 | 2.1 |
| Nigeria | 15 | 1 | 1.4 | 1.0 | 2.2 |
| Nigeria | 20 | 0 | 3.6 | 1.9 | 2.7 |
| Nigeria | 20 | 1 | 2.3 | 0.8 | 3.0 |
| Nigeria | 25 | 0 | 3.8 | 2.9 | 4.0 |
| Nigeria | 25 | 1 | 3.4 | 2.0 | 3.7 |
| Nigeria | 30 | 0 | 4.1 | 3.8 | 5.5 |
| Nigeria | 30 | 1 | 4.5 | 2.8 | 4.5 |
| Nigeria | 35 | 0 | 6.1 | 4.6 | 6.3 |
| Nigeria | 35 | 1 | 4.5 | 3.9 | 5.6 |
| Nigeria | 40 | 0 | 5.9 | 5.4 | 6.1 |
| Nigeria | 40 | 1 | 4.4 | 5.6 | 6.4 |
| Nigeria | 45 | 0 | 6.6 | 6.0 | 5.8 |
| Nigeria | 45 | 1 | 7.2 | 6.6 | 7.1 |
| Nigeria | 50 | 0 | 4.7 | 6.4 | 6.3 |
| Nigeria | 50 | 1 | 7.5 | 7.8 | 8.1 |
| Nigeria | 55 | 0 | 4.7 | 7.6 | 8.1 |
| Nigeria | 55 | 1 | 8.5 | 10.0 | 9.9 |
| Swaziland | 15 | 0 | 1.7 | 0.9 | 0.9 |
| Swaziland | 15 | 1 | 0.4 | 1.0 | 1.0 |
| Swaziland | 20 | 0 | 3.7 | 0.6 | 3.5 |
| Swaziland | 20 | 1 | 1.8 | 0.8 | 2.0 |
| Swaziland | 25 | 0 | 12.6 | 2.7 | 11.5 |
| Swaziland | 25 | 1 | 4.8 | 1.5 | 5.4 |
| Swaziland | 30 | 0 | 17.2 | 9.4 | 19.9 |
| Swaziland | 30 | 1 | 12.4 | 4.2 | 11.2 |
| Swaziland | 35 | 0 | 16.1 | 16.8 | 21.0 |
| Swaziland | 35 | 1 | 23.1 | 10.5 | 15.9 |
| Swaziland | 40 | 0 | 19.2 | 17.1 | 15.6 |
| Swaziland | 40 | 1 | 28.7 | 16.3 | 16.9 |
| Swaziland | 45 | 0 | 13.9 | 13.7 | 9.3 |
| Swaziland | 45 | 1 | 29.2 | 17.8 | 14.7 |
| Swaziland | 50 | 0 | 17.3 | 10.4 | 6.2 |
| Swaziland | 50 | 1 | 22.9 | 16.1 | 12.0 |
| Swaziland | 55 | 0 | 14.0 | 8.9 | 6.2 |
| Swaziland | 55 | 1 | 14.6 | 12.6 | 11.0 |
| Tanzania | 15 | 0 | 0.7 | 1.1 | 1.3 |
| Tanzania | 15 | 1 | 0.9 | 1.0 | 1.6 |
| Tanzania | 20 | 0 | 2.4 | 1.7 | 1.9 |
| Tanzania | 20 | 1 | 2.1 | 1.5 | 2.1 |
| Tanzania | 25 | 0 | 3.0 | 2.2 | 3.6 |
| Tanzania | 25 | 1 | 2.5 | 2.2 | 2.9 |
| Tanzania | 30 | 0 | 3.1 | 3.7 | 5.8 |
| Tanzania | 30 | 1 | 3.8 | 3.1 | 4.3 |
| Tanzania | 35 | 0 | 4.4 | 7.0 | 7.4 |
| Tanzania | 35 | 1 | 4.8 | 5.6 | 5.8 |
| Tanzania | 40 | 0 | 3.5 | 7.0 | 7.1 |
| Tanzania | 40 | 1 | 8.6 | 6.5 | 6.9 |
| Tanzania | 45 | 0 | 1.7 | 7.4 | 5.9 |
| Tanzania | 45 | 1 | 8.5 | 8.5 | 7.2 |
| Tanzania | 50 | 0 | 3.7 | 8.0 | 5.3 |
| Tanzania | 50 | 1 | 8.0 | 8.6 | 7.4 |
| Tanzania | 55 | 0 | 3.0 | 8.6 | 6.0 |
| Tanzania | 55 | 1 | 6.1 | 11.1 | 8.2 |
| Uganda | 15 | 0 | 1.0 | 1.1 | 1.7 |
| Uganda | 15 | 1 | 1.7 | 1.1 | 2.0 |
| Uganda | 20 | 0 | 2.9 | 1.6 | 2.1 |
| Uganda | 20 | 1 | 3.5 | 1.3 | 2.4 |
| Uganda | 25 | 0 | 2.9 | 2.2 | 3.8 |
| Uganda | 25 | 1 | 3.2 | 1.9 | 3.2 |
| Uganda | 30 | 0 | 4.9 | 3.9 | 6.2 |
| Uganda | 30 | 1 | 5.5 | 2.7 | 4.5 |
| Uganda | 35 | 0 | 5.9 | 5.7 | 8.1 |
| Uganda | 35 | 1 | 7.8 | 4.0 | 6.2 |
| Uganda | 40 | 0 | 6.8 | 6.8 | 8.8 |
| Uganda | 40 | 1 | 10.9 | 6.6 | 7.5 |
| Uganda | 45 | 0 | 3.5 | 7.7 | 7.8 |
| Uganda | 45 | 1 | 13.2 | 8.6 | 8.3 |
| Uganda | 50 | 0 | 5.5 | 7.7 | 6.6 |
| Uganda | 50 | 1 | 7.3 | 8.6 | 8.6 |
| Uganda | 55 | 0 | 6.8 | 8.0 | 6.9 |
| Uganda | 55 | 1 | 8.6 | 10.2 | 9.4 |
| Zambia | 15 | 0 | 0.5 | 1.3 | 2.0 |
| Zambia | 15 | 1 | 1.9 | 1.3 | 2.2 |
| Zambia | 20 | 0 | 1.7 | 1.6 | 3.1 |
| Zambia | 20 | 1 | 2.9 | 1.2 | 2.9 |
| Zambia | 25 | 0 | 4.7 | 2.9 | 6.3 |
| Zambia | 25 | 1 | 2.2 | 2.0 | 4.7 |
| Zambia | 30 | 0 | 5.2 | 5.4 | 10.6 |
| Zambia | 30 | 1 | 7.1 | 3.2 | 7.4 |
| Zambia | 35 | 0 | 8.8 | 8.2 | 13.7 |
| Zambia | 35 | 1 | 9.9 | 6.3 | 10.6 |
| Zambia | 40 | 0 | 5.7 | 10.5 | 12.9 |
| Zambia | 40 | 1 | 8.5 | 9.4 | 12.6 |
| Zambia | 45 | 0 | 6.0 | 10.1 | 9.6 |
| Zambia | 45 | 1 | 14.9 | 10.9 | 12.5 |
| Zambia | 50 | 0 | 13.6 | 8.5 | 7.3 |
| Zambia | 50 | 1 | 11.7 | 11.5 | 11.4 |
| Zambia | 55 | 0 | 5.9 | 8.6 | 7.6 |
| Zambia | 55 | 1 | 14.9 | 12.7 | 11.1 |
| Zimbabwe | 15 | 0 | 1.7 | 1.4 | 1.3 |
| Zimbabwe | 15 | 1 | 1.5 | 1.5 | 1.6 |
| Zimbabwe | 20 | 0 | 1.9 | 1.3 | 2.2 |
| Zimbabwe | 20 | 1 | 1.7 | 1.5 | 1.9 |
| Zimbabwe | 25 | 0 | 6.5 | 2.7 | 8.4 |
| Zimbabwe | 25 | 1 | 4.2 | 2.2 | 4.7 |
| Zimbabwe | 30 | 0 | 14.9 | 8.8 | 18.5 |
| Zimbabwe | 30 | 1 | 13.5 | 4.4 | 11.5 |
| Zimbabwe | 35 | 0 | 21.3 | 18.3 | 25.0 |
| Zimbabwe | 35 | 1 | 17.2 | 9.1 | 19.9 |
| Zimbabwe | 40 | 0 | 17.6 | 26.3 | 22.3 |
| Zimbabwe | 40 | 1 | 26.6 | 23.9 | 24.8 |
| Zimbabwe | 45 | 0 | 16.0 | 26.1 | 14.2 |
| Zimbabwe | 45 | 1 | 23.3 | 36.8 | 23.7 |
| Zimbabwe | 50 | 0 | 15.5 | 19.5 | 8.1 |
| Zimbabwe | 50 | 1 | 21.0 | 24.4 | 18.2 |
| Zimbabwe | 55 | 0 | 15.0 | 11.5 | 6.2 |
| Zimbabwe | 55 | 1 | 26.4 | 22.1 | 13.0 |
